# A new method for detecting signal regions in ordered sequences of real numbers, and application to viral genomic data

**DOI:** 10.1371/journal.pone.0195763

**Published:** 2018-04-13

**Authors:** Julia R. Gog, Andrew M. L. Lever, Jordan P. Skittrall

**Affiliations:** 1 Department of Applied Mathematics and Theoretical Physics, University of Cambridge, Cambridge, United Kingdom; 2 Department of Medicine, University of Cambridge, Cambridge, United Kingdom; 3 Department of Medicine, National University of Singapore, Singapore, Singapore; 4 Cambridge University Hospitals NHS Foundation Trust, Cambridge, United Kingdom; Keele University Faculty of Natural Sciences, UNITED KINGDOM

## Abstract

We present a fast, robust and parsimonious approach to detecting signals in an ordered sequence of numbers. Our motivation is in seeking a suitable method to take a sequence of scores corresponding to properties of positions in virus genomes, and find outlying regions of low scores. Suitable statistical methods without using complex models or making many assumptions are surprisingly lacking. We resolve this by developing a method that detects regions of low score within sequences of real numbers. The method makes no assumptions *a priori* about the length of such a region; it gives the explicit location of the region and scores it statistically. It does not use detailed mechanistic models so the method is fast and will be useful in a wide range of applications. We present our approach in detail, and test it on simulated sequences. We show that it is robust to a wide range of signal morphologies, and that it is able to capture multiple signals in the same sequence. Finally we apply it to viral genomic data to identify regions of evolutionary conservation within influenza and rotavirus.

## Introduction

In this paper, we present a new method for detecting signals in an ordered sequence of numbers. Here, a signal is a run in the sequence where the values tend to be unusually low. The development of this method was motivated by the need to identify ‘regions of interest’ in viral genomic data wherein methods are already established for assigning a score to individual codon positions [1]. Such scores indicate high or low variation between aligned sequences (equivalently low or high conservation between sequences), while taking into account codon usage across the genome and amino acid usage per site. Individual low or high scores are not typically of interest in themselves, but regions with many low scores might be important. These could represent the shadow of some additional feature that must be relatively conserved through evolution, such as a *cis*-acting signal or could indicate some sequence dependent element in an alternative reading frame.

In previous work seeking packaging signals in influenza A virus, the general order of magnitude of the length of such a region was a given input assumption. Thereafter a relatively simple method of using a moving average over an appropriate width could be used together with appropriate statistics to detect the presence of such a region. Despite its obvious limitations this approach has proven valuable, leading to identification of parts of the viral genome that warrant further investigation with additional theoretical methods or by chemical structural analysis [1–4]. However, this existing approach is limited in two important ways: the scale of the moving average is needed as an input, and the location of the signals is determined by eye. There might be more general applications in viral genomics, and beyond, if (i) it were not necessary to identify the size of the region *a priori* and (ii) the location of the region could be specified by algorithm and (iii) it were possible to disentangle multiple regions from within the same dataset.

At its most general, the problem is one of determining whether one or more runs of significantly lower numbers exist in a sequence of real numbers, and if such runs do exist, how much lower than the background sequence each run is and where each run is located. Expressed in this way, the problem is equivalent to that of extracting arbitrary signals of different lengths and magnitudes from a noisy background. Many types of approach exist, chiefly methods in image processing, including region growing algorithms, sliding window approaches and machine learning approaches. However none of these was found to meet all of our requirements, in particular many fall short in needing *a priori* information on the size of the signal to be found—we return to this point in the discussion. We have developed a method which meets all of our requirements, and we present the algorithm in general form below in the hope that it may be of use in broader applications than viral genomics. We have extensively benchmarked the method using simulated signals, and then applied our new methods to datasets from influenza and rotavirus.

## Methods

### Recast as random walk

We draw upon ideas used in an innovative method for detecting genetic recombination [5]. In that context the goal is to assign regions to different parental genomes in a parsimonious way. While not the same as the present problem, some elements are shared. In Boni *et al*. [5] a potentially recombinant sequence was turned into a sequence of steps up and down of size 1, and their cumulative sums considered as a random walk. Regions were then assigned to possible parental sequences by finding the large ascents and descents. By noting the total number of steps up and down, a given run can be checked to see whether it is more extreme than expected by random fluctuations. We extend this approach for the problem in hand. Our first additions are that (a) the step sizes may take any real value (not only plus or minus one), but with mean zero and variance one *and* (b) only the descents are of interest. A related approach is taken in gene set enrichment analysis [6] where the step sizes may take any real value but it is the maximum excursion from zero that is of interest.

Start with an ordered sequence of *n* real values *x*_1_, …, *x*_*n*_. For the applications below, they are the ordered scores for each codon in some gene, though these can be used for any application where a region of unusually low score would be of interest (throughout this paper, high scores could be found similarly by multiplying the *x*_*i*_ by −1). Next, linearly transform the *x*_*i*_ to ${\hat{x}}_{i}$ such that they have mean zero and variance one (preserving orientation): these are to be the step sizes in the random walk. Next, let *c*_0_, …, *c*_*n*_ be the partial cumulative sums, formed as follows:

$$\begin{array}{c} c_{0}=0\;,\;c_{k}=\sum_{i=1}^{k}{\hat{x}}_{i}\;\text{for}\;1\leq k\leq n. \end{array}$$

These *c*_*i*_ give a path that we will consider terms of a random walk.

#### Suitable test statistics: Max *Z*

The difference between any two cumulative sums *c*_*i*_ and *c*_*j*_ give the total of ${\hat{x}}_{i+1},\ldots {\hat{x}}_{j}$ (for *i* < *j*). The number of terms is *j* − *i*. If these $\hat{x}$ are assumed to be a random sample of all of the $\hat{x}$, then appealing to the central limit theorem then we would expect *c*_*j*_ − *c*_*i*_ to be approximately normally distributed with mean zero and variance *j* − *i*, for sufficiently large *j* − *i*. Hence this suggests the test statistic of the extremeness of a descent from *c*_*i*_ to *c*_*j*_ to be:

$$\begin{array}{c} Z_{ij}=\frac{c_{i}-c_{j}}{\sqrt{j-i}}\;\text{for}\;0\leq i<j\leq n \end{array}$$

Note the sign of the numerator is chosen so that descents give positive values. Finally, we take the maximal value of *Z*, over all possible start and end positions:

$$\begin{array}{c} Z=\max_{i,j:i<j}Z_{ij} \end{array}$$

Note, the range *i*, *j* that maximises *Z*_*ij*_ might not correspond to the largest descent in terms of the raw difference (*c*_*j*_ − *c*_*i*_) alone: the denominator will penalise wide ranges.

### Improvements in computational speed in finding max *Z*

The simplest way to find the most extreme *Z* is to search over all possible *i*, *j* combinations. However, this is computationally expensive for long sequences. In particular, as many bootstrap recalculations are necessary (see below), this algorithm will be used many times so any improvement in speed here will be valuable. Here, we outline some ideas that are useful to achieve this.

The key is to identify qualities that must be satisfied by the *i* and *j* corresponding to the largest *Z*_*ij*_. This particular descent must start at its highest point and end at its lowest point. If either of these conditions were not met it would be possible to trim the interval to an intermediate extremal point and have an equal or larger drop (*c*_*i*_ − *c*_*j*_) and a shorter width (*j* − *i*), and thus a more extreme *Z*. So in other words, if *i* and *j* are the end points corresponding to the most extreme *Z* (i.e. *Z* = *Z*_*ij*_) then for all *k* such that *i* < *k* < *j* we must have *c*_*i*_ > *c*_*k*_ > *c*_*j*_.

This can be exploited in algorithm design by simply noting that the first and last steps must be downwards, hence ${\hat{x}}_{i+1}<0$ and ${\hat{x}}_{j}<0$. This immediately limits the set of potential start and end points:

$$\begin{array}{ccc} \text{Potential}\;\text{starts} & = & \left\{i\;:\;\text{such}\;\text{that}\;{\hat{x}}_{i+1}<0\right\}=s_{1},s_{2}\ldots s_{N},\\ \text{Potential}\;\text{ends} & = & \left\{j\;:\;\text{such}\;\text{that}\;{\hat{x}}_{j}<0\right\}=e_{1},e_{2},\ldots e_{N}, \end{array}$$

labelling each group with the *s*. and *e*. in increasing order. Note that *s*_*u*_ = *e*_*u*_ − 1. This will already reduce the number of possible *i*, *j* combinations to check, as the only possibilities are now *i* = *s*_*u*_ and *j* = *e*_*v*_ for some *u*, *v* with *u* ≤ *v*.

The same quality (no extremal point within the descent) may be used for further speed improvement. Suppose we have the optimal descent with start *i* and end *j*. If we have some *m* < *j* such that *c*_*m*_ ≤ *c*_*j*_ then *m* cannot be inside the descent, so we must have *m* < *i*. This can be used to further limit the possible *i*, *j* pairings to check. Here we work based around each potential end location and find a way to limit which potential start locations may be associated with it by looking for a suitable value to play the role of *m*.

For each potential end *e*_*v*_, find the potential end most immediately before which has equal or lower *c* (or zero if none). More formally:

$$\begin{array}{c} f\left(v\right)=\text{max}\;(\{w\;:\;w<v\;\text{and}\;c_{e_{w}}\leq c_{e_{u}}\}\;,\;0)\;. \end{array}$$

If *f*(*v*) = *w*, we can deduce that the end point *e*_*v*_ cannot be optimally paired with any start *i* with *i* < *e*_*w*_. This rules out all *s*_*u*_ for *u* ≤ *w* (as *s*_*u*_ = *e*_*u*_ − 1 < *e*_*u*_ ≤ *e*_*w*_).

The *f*(*v*) can themselves be computed efficiently by working iteratively. Start by noting *f*(1) = 0 (there is no end point before the first one). Then work through increasing *v*. For each *v*, we sequentially trial different candidates *w* for *f*(*v*) = *w* until we find the first one that meets the condition $c_{e_{w}}\leq c_{e_{v}}$. First try *w* = *v* − 1. If the condition is met then stop. Otherwise iterate the trial *w* by the function *f*, i.e. new *w* = *f*(old *w*). This manoeuvre helps as we skip the in between values where by the definition of *f* we know they do not correspond to lower ends, and thus will not meet the condition. Repeat this checking and iterating with *f* until either a *w* is found that meets the condition or we have reached *w* = 0: either way accept this value so that we have now found *f*(*v*) = *w*. Continue to the next *v*.

Now for a given end point *j* = *e*_*v*_, we need check only the starts *i* = *s*_*u*_ with *f*(*v*) < *u* ≤ *v*. We found this to give a substantial improvement in the time taken to find the maximal *Z*: this is particularly valuable in the bootstrap computations below.

Instead of limiting which start points are feasible with a given end point, we could equivalently have used the same approach to limit which end points are feasible with a given start point. One might consider using both approaches and then take the intersection of which pairs to try. We found however that this double approach did not lead to any further speed improvements in practice for the work presented here, but it may warrant further consideration in different settings, such as extremely long sequences.

### Statistical testing

For a single value of *Z*_*ij*_, for large difference between *i* and *j*, we might be able to appeal to the central limit theorem and normality to determine the significance. However here we are taking the largest of many non-independent values, hence a bootstrap approach is used for significance calculations.

The *x*_*i*_ (or equivalently the ${\hat{x}}_{i}$) are permuted and the new maximal *Z* is computed for this sequence. This is repeated many times to generate a large set of resampled *Z*. The real *Z* is then compared with this set of resampled *Z*. If the real *Z* is ‘beaten’ by fewer than 1% (or 5%) of the resamples, then the region is deemed to be significant with *p* < 0.01 (or *p* < 0.05).

For the benchmarking results below using the simulated datasets the *Z* are resampled 1000 times. For the virus datasets this is increased to 10,000 resamples. For some of the plots below results are shown for both 1% and 5% significance levels. But if a single level is used it should be taken as 5% below unless otherwise stated.

### Detecting multiple signals

It is possible that there is more than one significantly low region in the *x*_*i*_. These could be separate and the weaker signal could be initially masked while the algorithm picks up the stronger one. Alternatively, signals could be nested and the algorithm above may pick up a stronger core region, whereas there is still a significant but weaker broader region. We actually do not see this second possibility in any of the viral data below, but we believe it could be relevant in broader usage.

To extend our method to find multiple signals in either case, if one significant region is found (from the process described above), then the corresponding *x*_*i*_ are simply excised and the remainder concatenated. The full process is then repeated from the start (including normalising to get the $\hat{x}$ and a fresh round of bootstrapping to test the significance of the largest *Z*). This should be repeated until no further significant region is found.

### Implementation

Two versions of code to run our methods are given in S1 File (Mathematica) and S2 File (Python). While the methods are fully specified by the mathematical description above and should be amenable to quick implementation in users’ preferred language to interface to other code, this sample code may be useful for trialling the method. A detailed worked example with a small dataset is given in S1 Appendix. This gives the values for the *x*_*i*_ explicitly and runs through to graphical output, and also illustrates the methods for computational speed improvements described above.

The numerical results below were produced in Mathematica (Wolfram Research, versions 10 and 11). The virus datasets used are given in S1 Dataset.

## Results

### Benchmarking the method: Application to simulated signals

To show our method in action, and to test how effective it is at finding signals of various forms and strengths (i.e. different morphologies and numerical deviations from the background number sequence of a lower subsequence of numbers), we simulate sets of *x*_*i*_ that contain some mock signal, and then attempt to recapture the signal using the algorithm described above. The first section below shows the application of the algorithm step by step to a single dataset in order to illustrate the method. The next section shows many simulated and attempted recaptures of signals of the same dimensions but varying signal strength. Subsequent sections show detection success rates with varying signal strength, but also with the simulated signal changed in width, or with changes in the total sequence length, reduced signal density, or a second signal added. These demonstrations show that our methods are able to find a wide variety of signal types, but also cautions about cases where a signal may be missed.

#### Worked example, single simulated dataset

With viral informatics in mind, we take our default simulated signal to be a region of length 100 in the middle of a sequence of length 500. These lengths are chosen to be loosely representative of a large conserved region (e.g. a conserved structural motif) within a viral gene. Here, the *i* represents amino acid position and the real number score *x*_*i*_ is some measure of genetic conservation (e.g. as in [1]). We use these dimensions as an example, but below we vary these characteristics to determine how well the method can pick up signals in other forms.

**Fig 1:**
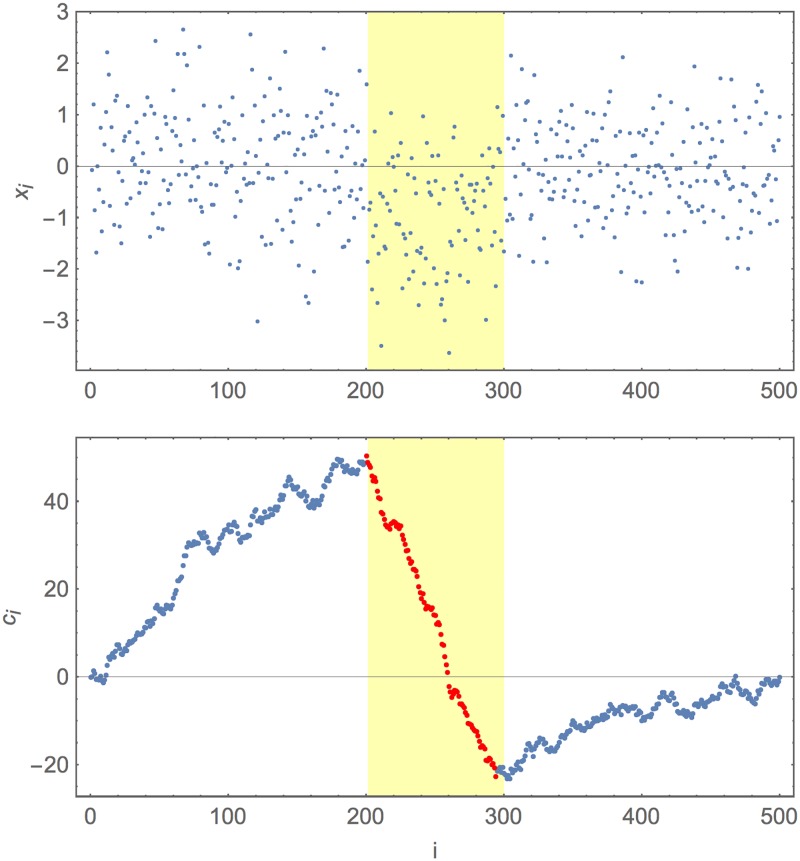
Example of a simulated and recaptured signal. The upper plot gives simulated *x*_*i*_ for a ‘gene’ of length 500, with a signal region length 100 in the middle. The background *x*_*i*_ are simulated as independently normally distributed with mean zero and variance 1, the signal positions are independently normally distributed with mean −1 and variance 1. The lower plot gives the *c*_*i*_ for this same simulation: the normalised cumulative walk. In both panels the simulated signal region is the highlighted region (from positions 201 to 300 inclusive). In the lower figure the maximum *Z* descent is marked in red. The location of the maximal descent corresponds well to the simulated signal.

**Fig 2:**
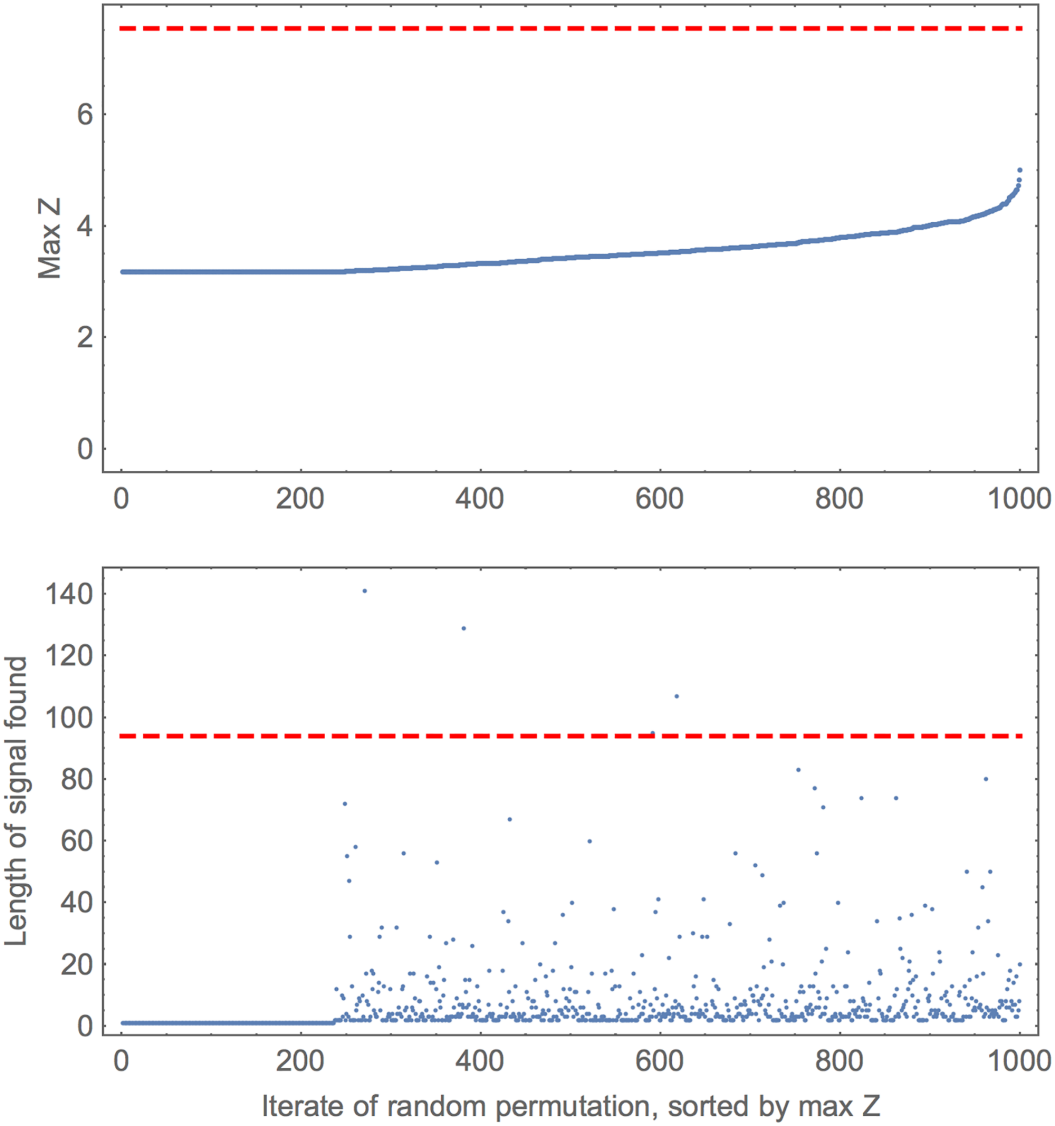
Example of a simulated and recaptured signal. The example is exactly as in the previous figure but here some statistics from many random permutations of the *x*_*i*_ are shown. For illustration 1000 such permutations are presented and these are represented along the horizontal axis. For clarity these are sorted by their maximum *Z*. The upper plot gives the max *Z* for each run (blue dots). The lower plot gives the length of the signal found. In both, the red dashed line gives corresponding ‘real’ value (from the simulated data in original order). The real max *Z* is much higher than any *Z* from the permutations: we say 0 out of 1000 bootstrap *Z* were higher. The plateau in each panel is discussed in the main text.

All the *x*_*i*_ are independently sampled from normal distributions with variance one and mean zero for the background (everything except the simulated signal) and mean −1 for the simulated signal. Having the *x*_*i*_ with mean shifted by one standard deviation seems to be a strong signal, and a single example is shown here to illustrate the method in action: see Figs 1 and 2. The absolute values of mean and variance are not important as the *x* will be linearly rescaled to give the $\hat{x}$.

In Fig 1 it is interesting that, while in the upper plot (the raw *x*_*i*_) the signal can perhaps be discerned by a careful eye, there is no doubt at all to the observer that there is a clearly non-random descent in the middle of the lower plot. To the human eye the cumulative plot is already much easier to read than the scattered points alone. This insightful development of Boni *et al*. [5] is key to our method here.

Fig 2 shows the outputs from the bootstrapped sequences (permutations of the original *x*_*i*_, as described above). This shows that the maximum *Z* found in the original *x*_*i*_ is clearly above that from any permutation. In both panels of Fig 2 there is a plateau on the left, corresponding to the maximum *Z* in those permutations coming from a single value of *x* (which will be the minumum of the *x*_*i*_). As this single value is present somewhere in all permutations, the maximal *Z* must be greater than or equal to this value. After that, the maximum *Z* comes from variable sized regions: these are often very short, but occasionally long, and indeed sometimes longer than the ‘real’ region width detected.

This suggests a possible variation of the statistical approach: if signals of length 1 or below some certain width could be excluded as not being of interest at all, then these could be excluded in bootstrapping and the accepted maximum *Z* would be lower for all of those permutations. We have chosen to not do this here, instead taking the more conservative approach of allowing detected signals of any length to count towards the bootstrapped distribution for maximum *Z*.

#### Varying the strength of the simulated signal

Still using the pattern of a simulated signal in the middle 100 positions of a sequence of length 500, the strength of this signal is now varied. As above, all the *x*_*i*_ are drawn independently from a normal distribution with variance 1. The mean is zero for the background, and now the signal has its mean shifted downwards by some value; this value is the ‘strength’ of the signal. The above example thus had strength 1. Here strength is varied from 0 to 1 in steps of 0.1. For these and all the simulated signals below, 2000 simulated sequences are produced for each value of strength. We explore significance at 5% and 1% levels, corresponding to max *Z* being more extreme in less than 50 or 10 permutations out 1000 respectively. (The same runs were used for both significance levels by running the algorithm through to completion for the weaker significance and then subsequently checking for earlier termination under the stronger significance test).

**Fig 3:**
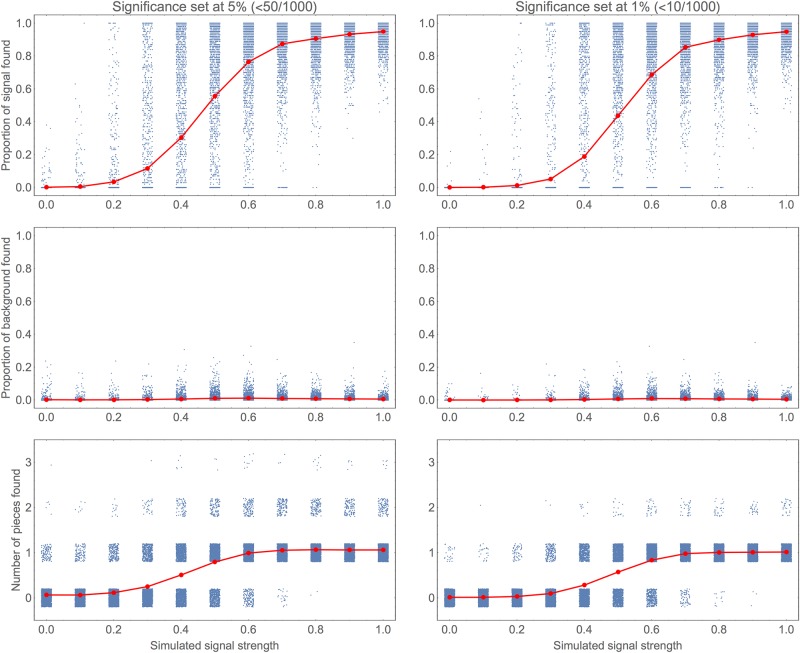
Effect of varying strength of simulated signal: Summary statistics. For a range of different simulated signal strengths 2000 simulated sequences were generated and the algorithm applied to attempt to recapture the signal. This figure gives some summary statistics from these. The left column corresponds to setting the required significance level to 5% and the right to 1%. In each subplot the individual simulations are shown as blue dots and their mean for a given value of signal strength shown in red. To show the density more clearly the dots have been dithered slightly: horizontally only in the upper two plots and vertically also in the lower plots. The upper panels give the proportion of the signal (the middle 100 of 500 positions) that was recaptured by the algorithm. The middle panels give the proportion of the 400 positions of background that is determined to be signal by the algorithm. The lowest panels give the number of separate pieces that are detected.

Fig 3 shows summary information for these simulations. The mean proportion of the signal that was recaptured transitions fairly steeply from 0 to around 1 as the signal strength is increased. However during the transition there is still wide variation: relatively weak signals can still sometimes be nearly fully recaptured and strong signals can have part of their region missed. The false positive proportion remains low throughout. The number of pieces detected is mainly zero for low strength, a mix of zero and one for intermediate strength and then one for the strongest simulated signals. Very occasionally two or more pieces are found: the extra pieces generally corresponding to small false positive fragments. Unsurprisingly there are more of these at 5% significance than at 1%.

**Fig 4:**
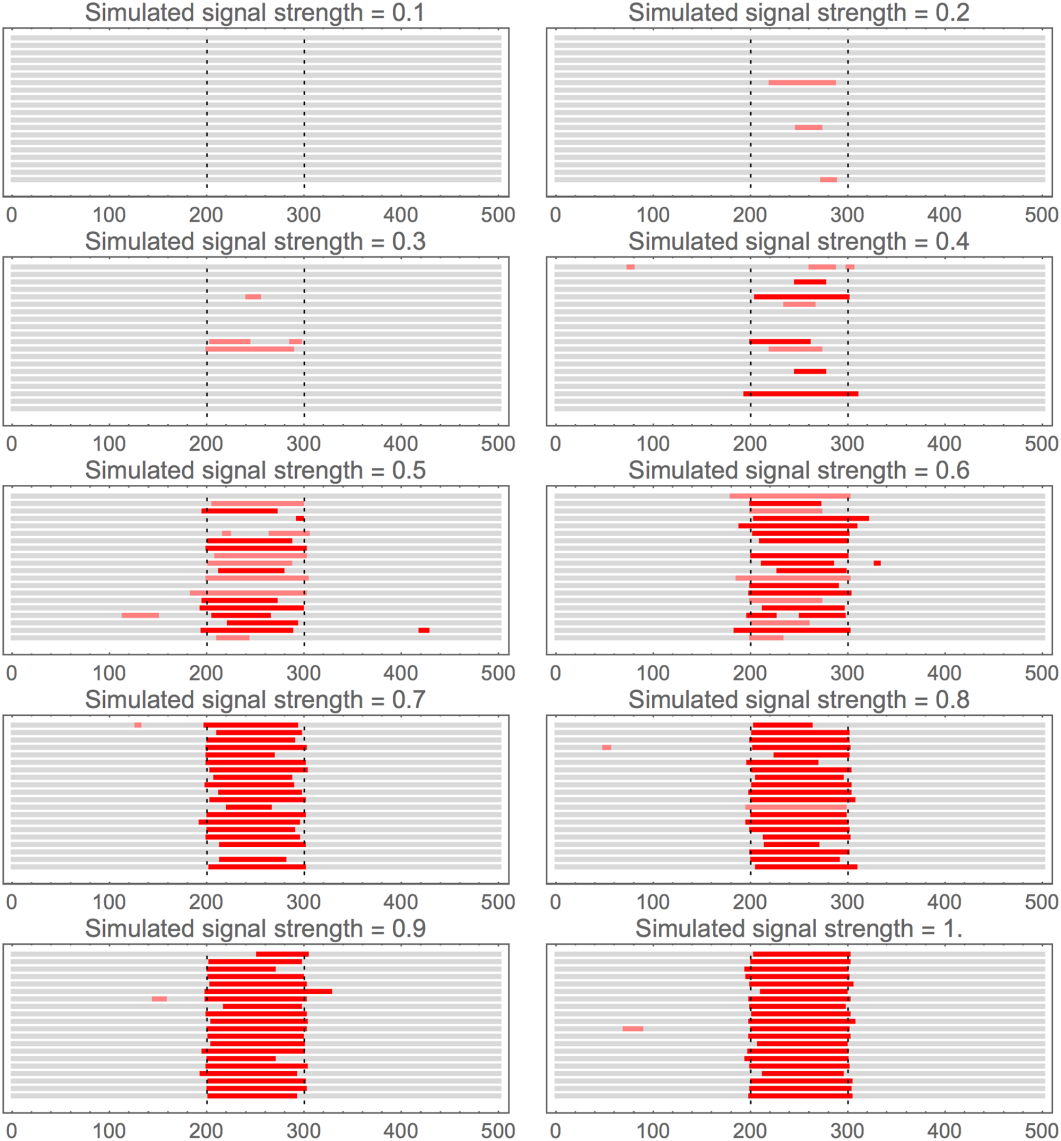
Effect of varying strength of simulated signal: Sample outputs. These are graphical representations of the regions found for simulated and recaptured signals. In the interests of space and clarity only a random set of 20 individual outputs is shown for each value of strength shown: they are arranged stacked vertically with no special ordering applied. If a region is detected it is shown as a horizontal bar: red if significant at 1% level, pink if only at 5% level.

Fig 4 shows a graphical representative output for a randomly selected sample of these simulations. Along with the previous figure, these demonstrate that the signal is usually either nearly fully recovered or not found at all; partial detection is only likely during the sharp transition in strength. The occasional false positive region tends to be very short: again if it were known that the regions sought were actually over a certain length this could be used to reduce the false positive rate, but here we aim to make as few assumptions as is possible.

#### Varying the morphology of the simulated signal and sequence

**Fig 5:**
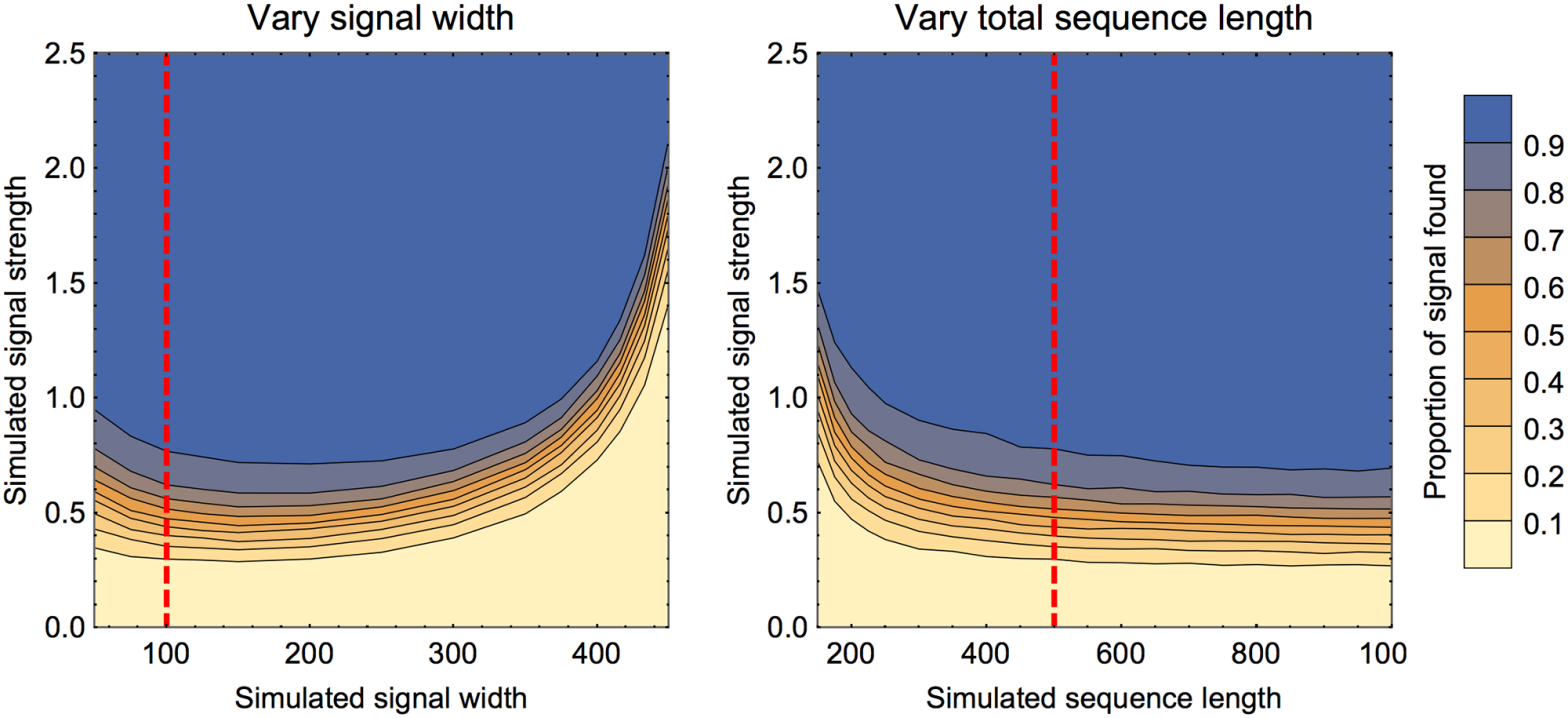
Effect of varying the width of the signal and length of the sequence. In both panels the colour of the region represents the mean proportion of signal recovered. Points were sampled along lines of fixed width or length to find the locations of the contours. In both panels the red line shows the transept that corresponds to the default set-up above (signal width 100, sequence length 500). The left panel shows the signal width varied from 50 to 450, with sequence length fixed at 500. The signal is placed in the centre of the sequence. Each contour is fairly horizontal in the range 100 to 300 and curves up at the ends. The curve up at the left shows that shorter signals are marginally harder to detect in that they must be stronger for the same success rate. The steep curve up at the right indicates that the signals are very hard to detect when they constitute the majority of the sequence. The right panel shows the sequence length varied from 150 to 1000 with signal width fixed at 100. The same effect is seen again: if the sequence length is not much longer than the signal, then the signal is relatively hard to detect. The contours are almost horizontal towards the right of the figure. Once the sequence is several times the signal length there is little further change.

The simulations above were of a signal of length 100 within a sequence of length 500. The algorithm is insensitive to the location of the signal (except when the signal is at one end of the sequence and hence it is not possible to overshoot the fitted region in one direction). We now vary the morphology of the signal in various ways. First we explore the effect of changing the width of the signal and the length of the full sequence: see Fig 5. In summary, the method performs well on a wide range of signal widths and sequence lengths, only breaking down in the extreme case where the sequence is mostly signal. This is unlikely to occur in applications to viral sequence analysis, but if it is a likely case in other applications the algorithm could be applied to inverted data (minus values) to find locations that are not signal: this is equivalent here say to comparing finding a signal of length 50 rather than 450.

**Fig 6:**
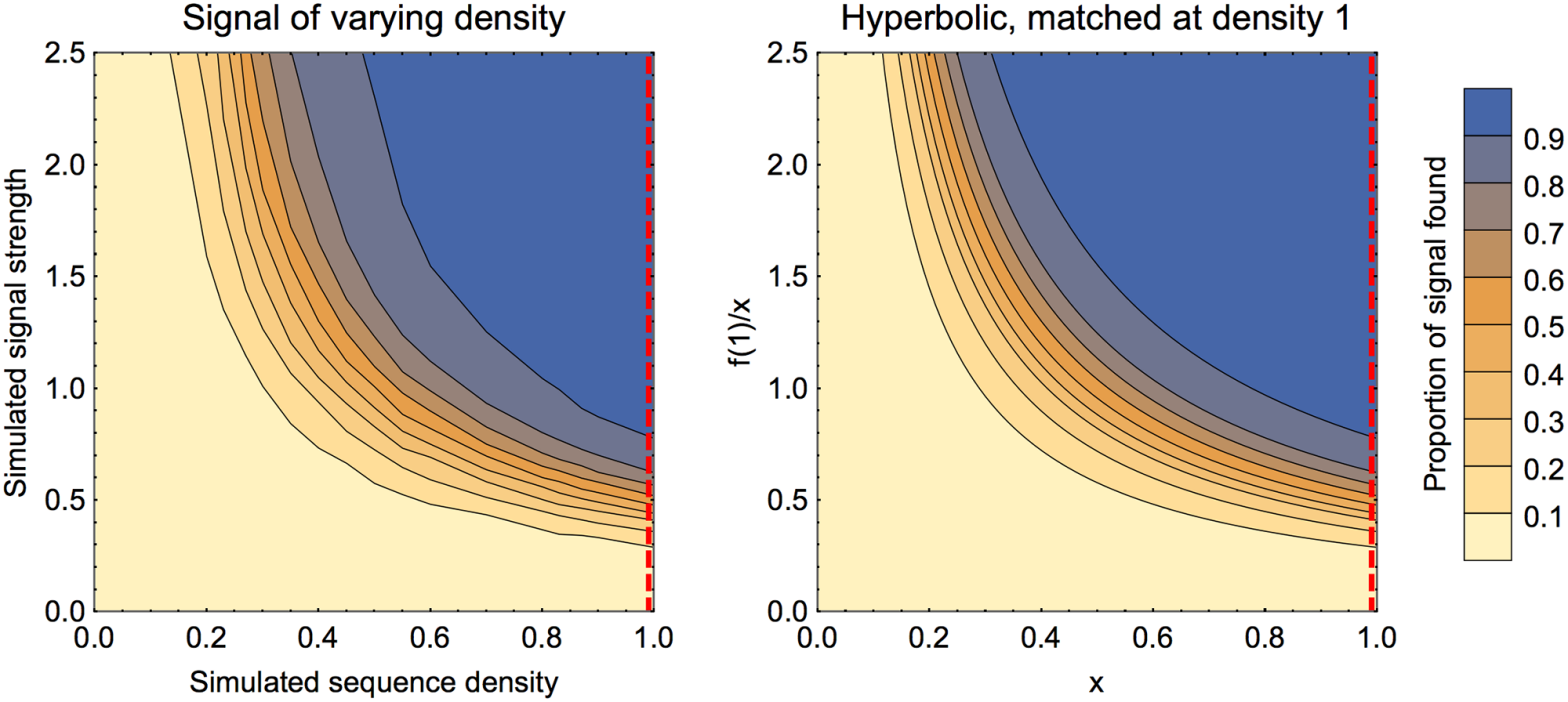
Effect of varying signal density. In the left panel the colour of the region represents the mean proportion of signal recovered. Points were sampled along lines of fixed density to find the locations of the contours. The panel on the right shows an extrapolation by taking the results for density one, and extending the contours proportionately to inverse density. In both panels, the red line shows the transept that corresponds to the default set-up above (density 1).

In addition to the lengths changing, it could also be that the signal is patchy. We explore this by allowing the density of the signal to vary. Here ‘density’ is used to mean the proportion of positions within the signal that have *x*_*i*_ with shifted mean. These positions are picked at random (subject to meeting the right proportion), and the remaining positions have *x*_*i*_ chosen as if they were background positions. These results for variable density are shown in Fig 6. Unsurprisingly, a reduction in signal density means that an increased signal strength is needed for the same success rate in recovering the signal. It might be asked if the required signal strength is simply inverse to density: this would roughly correspond to a line of fixed *average strength*, though it is slightly complicated by extra variance. Comparing the two panels in Fig 6 we see that this explains much of the pattern here: a low density signal behaves as if it were a proportionately lower strength signal. However, there are some differences, particularly seen by comparing the contour for 0.9 mean proportion recovered. This can be interpreted as the signals still being found at lower density, but the accuracy of finding their exact locations being compromised for the patchy signals. It seems unlikely that any method could evade this problem, so we caution that for very heterogeneous signals, the regions found will not be so precise.

**Fig 7:**
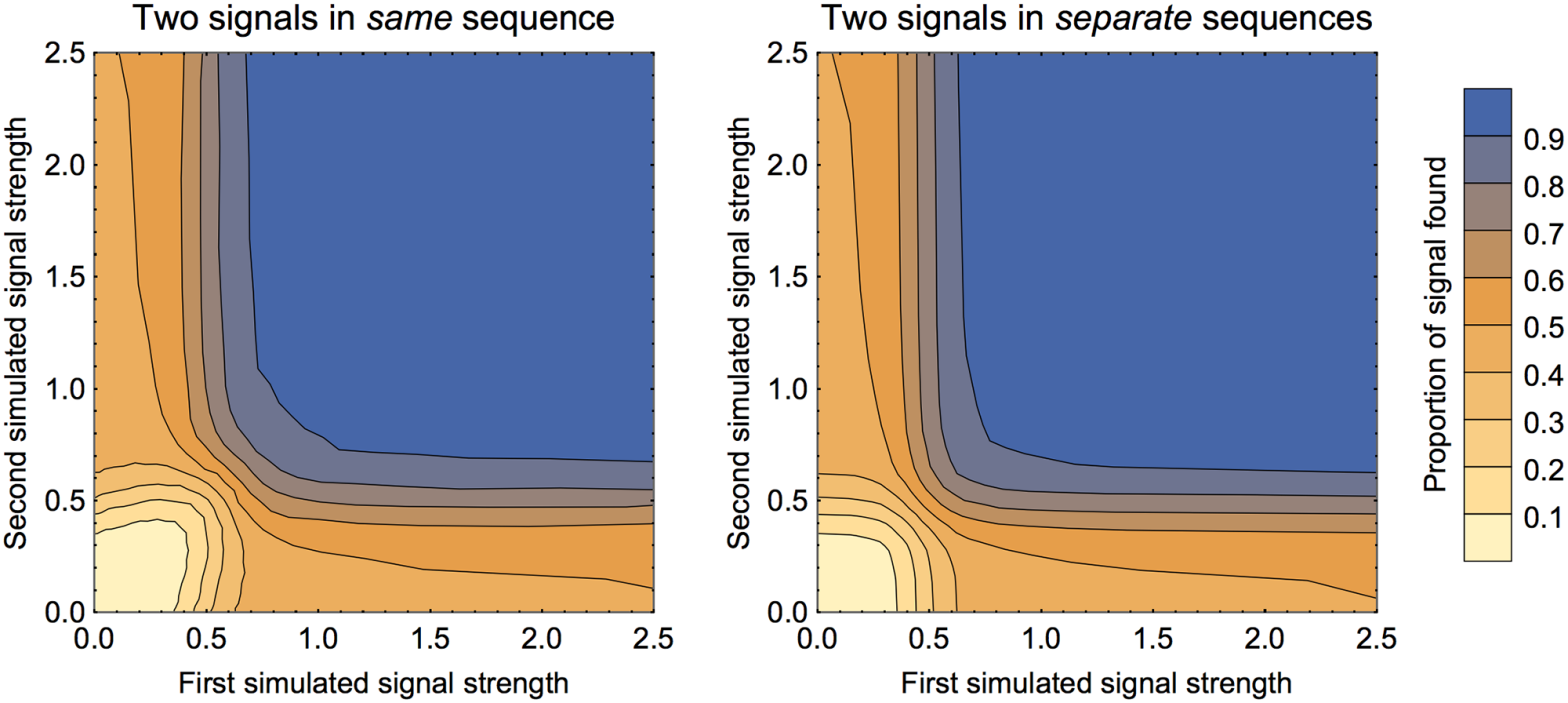
Finding two signals in same sequence, varying strengths. In both panels the colour of the region represents the mean proportion of signal recovered. The left panel shows the proportion of *total* signal found when there were two signals within the same sequence (spaced as 100 background, 100 signal one, 100 background, 100 signal two, 100 background). The points were sampled adaptively along lines of fixed signal ratio to find the locations of the contours (only half of the figure was generated in this way; the results were then reflected across the diagonal, in the interests of computational speed). The right panel shows a similar output for two signals in two separate sequences of length 500. As these computations for the two sequences are independent, this was generated using the results for a single signal of length 100 and extrapolating.

One of our stated aims for the method is to be able to detect multiple signals in the same sequence. We investigate that here by creating two separate signals in the same sequence. The results are shown in Fig 7 (left panel). As might be expected, the portion found is high when both signals are strong and low when both are weak. Also as expected, when one signal is strong and the other is weak, about half of the total signal is found (corresponding to finding one of the two signals). To see if the signals are interfering with the success of finding the each other, two signals in the same sequence can be compared with finding two signals in two separate sequences. As can be seen in Fig 7 (both panels), these are strikingly similar.

**Fig 8:**
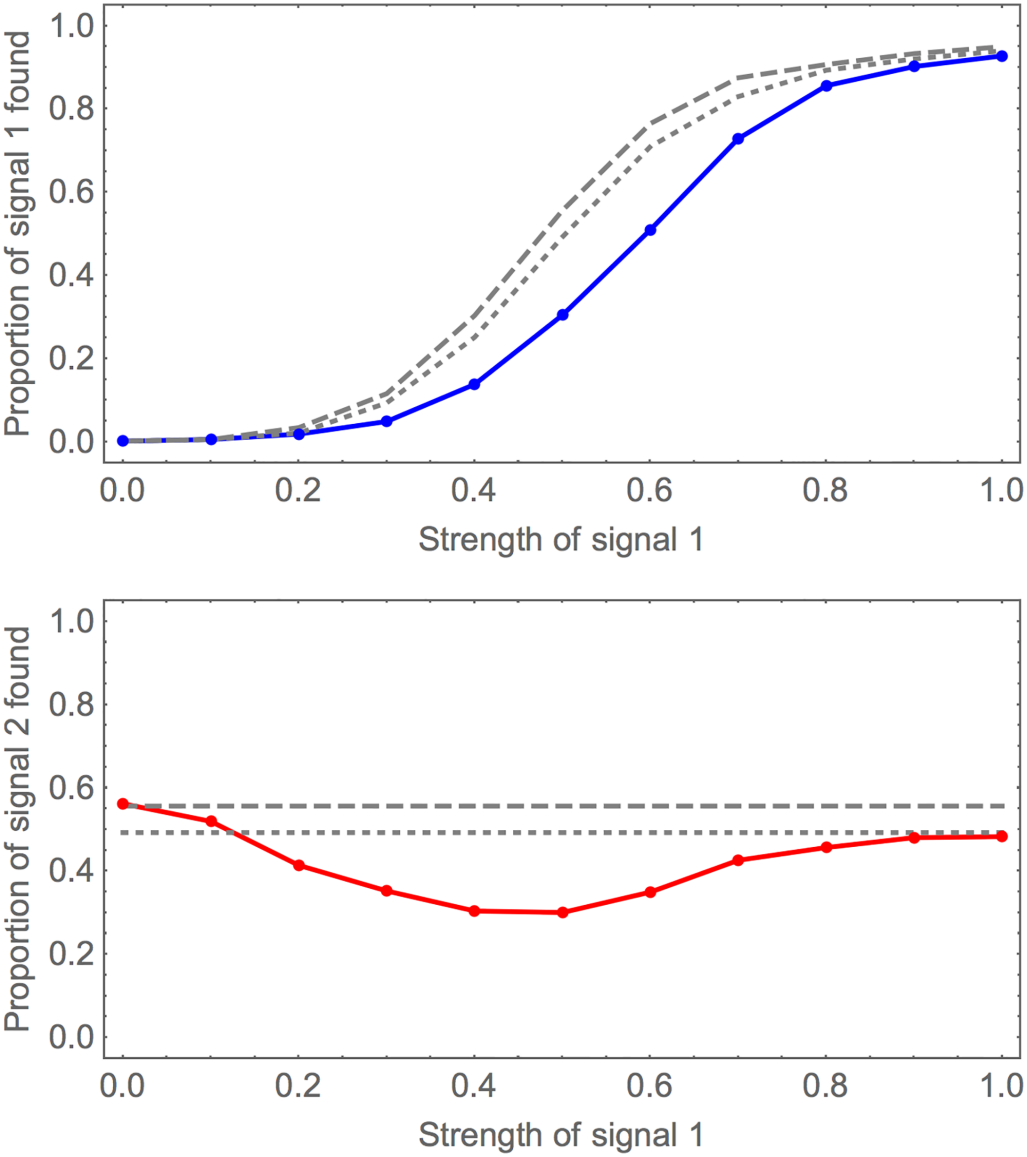
Finding two signals in same sequence, varying strength of one signal. Here we simulate two signals in the same sequence with the same arrangement as in the previous figure. The first signal’s strength is varied, the second signal’s strength is kept fixed at 0.5. The solid curve in the upper plot is the mean proportion recovered of signal 1 and the lower plot for signal 2 (dots for computed values, line segments to join). The grey dashed and dotted curves are for comparison with a single signal, either in a sequence of total length 500 (dashed) or 400 (dotted), with strength as signal 1 (upper plot) or signal 2 (lower plot). The first signal is still sigmoidal in its proportion found as a function of strength, but sits under the curves for equal strength signal alone (either in 400 or 500 length sequence). When the first signal strength is zero, the proportion of the second signal found matches that of a single signal of strength 0.5 in a sequence of length 500. When the first signal is strong the proportion of the second signal found is comparable to a single signal of strength 0.5 in a sequence of length 400. When the first signal is at intermediate strength, its effect of interference on the second signal is most clear.

In theory two signals in the same sequence should very marginally mask each other. When bootstrapping to check significance of one potential signal, the other signal will be included in the ‘background’ and hence shift the resampled scores closer to the first signal than if there were no other signals. Visually from Fig 7 the effect is hard to detect, so this is explored also in Fig 8. The effect is strongest when both signals are of an intermediate strength. For more than two signals, we expect these results to still hold: if there are signals that are strong enough to be nearly always found, then the other signals will be found with success comparable to the full sequence length being shorter (after the first signal is found and removed). We can see from the results above on sequence length that we can expect our method to be robust except in extreme cases such as the sequence consisting mainly of signals with little background. The most dangerous situation is that of multiple signals of intermediate strength, when they may mask each other so that no signal is found.

These benchmarking results suggest that our proposed method does indeed find signals of a wide range of morphology, and that multiple signals (or multipartite signals) can typically be found without masking each other. We have explored a range of factors here that we think are likely to be important for applications we can envisage, with a particular view to using these methods for viral sequence analysis. We hope our checks are suitably generic so that a wider range of applications will be covered. However if researchers plan to use this method for other applications where the default expected arrangement of signals is strikingly different from the ones imagined here, then it might be prudent to carry out a similar benchmarking exercise to the one conducted here, focussing on likely signal morphologies (arrangements of the low numbers within the overall sequence) for that specific application.

### Application: Viral genome data

Finally, to show our methods in use in a real application, we apply our algorithm to viral genomic data (now using 10,000 bootstrapped reshuffles for each gene). The data are from previously published works on influenza A [1] and rotavirus [4]. For each codon position, a score was calculated for the amount of variation between sequences (mean pairwise distance: MPD) and normalised taking into account amino acid usage and codon bias (full methods are presented in Gog *et al*. [1]). These scores are given in the first column for each gene in S1 Dataset.

The *x*_*i*_ as described above are set as these normalised MPD values with one further refinement. In the previous works, if both the observed MPD and the expected MPD were zero, the normalised MPD was set to equal 1. The rationale for this was simply that the value should indicate that the observed distribution was exactly as expected (indeed, it could not have been otherwise). This new method is able to cope with varying width scales so these sites can be deemed to be uninformative, and simply excluded. This means that the subscripts *i* of the *x*_*i*_ do not correspond exactly to codon position, as some uninformative sites are skipped in the sequence—this just requires some care with book-keeping when translating back to the location on the genome. The uninformative sites are indicated in the second column for each gene in S1 Dataset.

**Fig 9:**
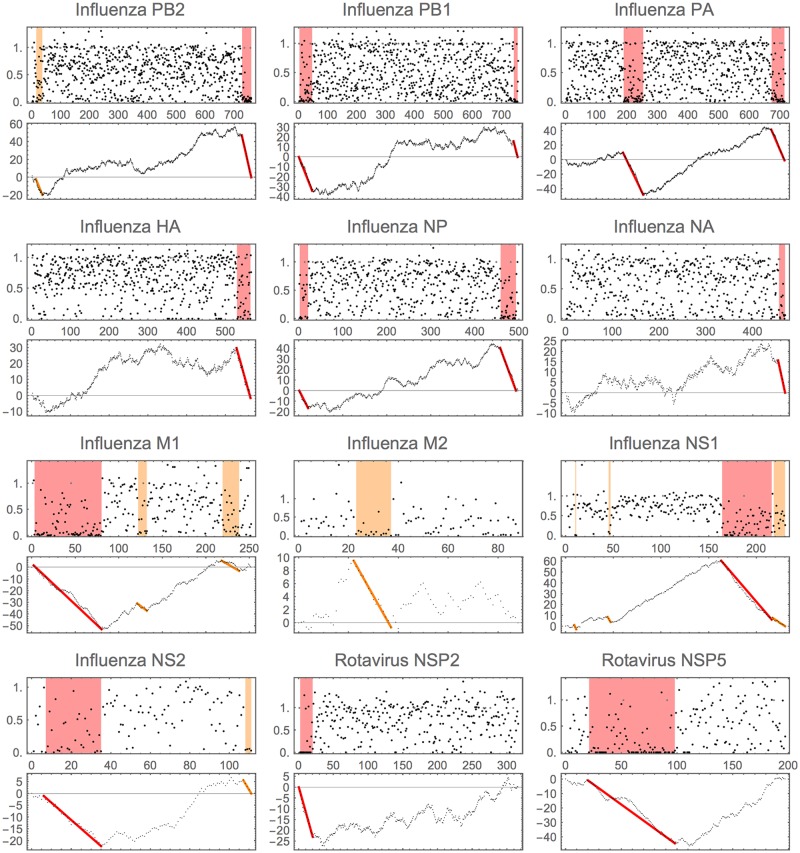
Regions in virus data. In each pair, the upper plot gives the normalised MPD (*x*_*i*_) with ignored values marked in grey. The lower plot gives the cumulative normalised values (*c*_*i*_). The coloured bars in each indicate significant regions found. Red indicates *p* < 0.01 and orange *p* < 0.05. Details of locations and order in which the regions were found within each gene are given in Table 1. Note that the cumulative plot is strictly only correct for the first region found. For subsequent regions the cumulative values considered were renormalised after the previous region(s) were removed. For clarity all regions are marked on the original cumulative plot.

Our results are shown graphically in Fig 9. Table 1 includes the exact locations, the order in which the regions are found by the algorithm, the number of reshuffles that were more extreme and the consequent significance level. Where pertinent previous work exists, Table 1 also includes a brief comment on a possible reason why the region may be under evolutionary constraint.

**Table 1:** Summary of regions found.

Virus & Gene	Order found	Location	P	Comment
Influenza PB2	2	15-36	* (188)	Packaging
Influenza PB2	1	727-759	** (0)	Packaging
Influenza PB1	1	2-46	** (0)	Packaging & overlap PB1-F2 [36, 37]
Influenza PB1	2	742-755	** (20)	Packaging
Influenza PA	1	189-253	** (0)	Overlap PA-X [13]
Influenza PA	2	672-714	** (0)	Packaging
Influenza HA	1	529-564	** (4)	Packaging
Influenza NP	2	2-21	** (82)	Packaging
Influenza NP	1	458-493	** (0)	Packaging
Influenza NA	1	456-469	** (59)	Packaging
Influenza M1	1	3-80	** (0)	see main text
Influenza M1	3	122-132	* (231)	see main text
Influenza M1	2	219-238	* (250)	see main text
Influenza M2	1	23-37	* (255)	see main text
Influenza NS1	4	10-11	* (216)	?
Influenza NS1	3	45-47	* (311)	?
Influenza NS1	1	164-216	** (0)	Overlap NS2
Influenza NS1	2	218-230	* (414)	Overlap NS2
Influenza NS2/NEP	1	7-35	** (15)	Overlap NS1
Influenza NS2/NEP	2	108-111	* (239)	?Packaging
Rotavirus NSP2	1	2-20	** (0)	See [4] and main text
Rotavirus NSP5	1	21-98	** (0)	Overlap NSP6, see [4]

The ‘order found’ refers to the sequence in which the regions were detected using our sequential algorithm. The location is given in codon position from the start of the ORF (this has accounted for the ‘uninformative positions’). Under P, ** indicates *p* < 0.01 and * indicates *p* < 0.05. The number in brackets is the number of the 10,000 bootstrapped resamples that were more extreme, giving a more nuanced measure of significance.

For the comments marked ‘packaging’, these regions are likely associated with *cis*-acting packaging signals in Influenza A; either previously identified or likely by analogy [1]. Comments also include where a region detected is likely associated with a shifted overlapping reading frame associated with an alternative gene product. For two regions in NS1, there is no immediately obvious parsimonious explanation. Given their short length and that their significance is around the 2 − 3% level, it might be that these are false positive. It would not be surprising to have one or two of these when analysing several different gene datasets.

To a large extent, many of the regions identified by this method correspond well to known features, such as packaging signals and overlapping but shifted reading frames of a second gene product from the same segment [1, 4]. However, the method also identifies regions that were not clear in previous analyses, notably some regions in the influenza glycoproteins HA and NA and perhaps also in the second gene products NS2/NEP that all appear to be analogous to packaging signals mapped in other segments.

The picture is rather complex for influenza segment 7 (results associated with M1 and M2 in Table 1), involving multiple possible gene products. The regions here correspond to shifted reading frames in some cases, but also splice donor or acceptor sites or other factors controlling expression of the alternative products [7, 8]. In some segments, the regions we detected correspond to conserved structural motifs [9, 10] including a number of RNA sequences in influenza that are known to undergo functionally important and extensive structural switches such as stem-loop to pseudoknot [11, 12]. The regions found in rotavirus correspond to those previously identified, including a region of conserved base pairing between the 5’ coding region and 3’ UTR of the ssRNA segment in RNA8 (NSP2) [4].

Our method would also have been useful in more clearly determining the large region inside influenza PA, and to have given a specific range for it much earlier than was identified. The analysis of 2007 [1] did explicitly mention this approximate region as potentially of interest, but the chosen short moving average window (tuned for packaging signals) missed its strength. This region later was found to be functionally important in evading host immune response (PA-X, [13]).

This success in identifying regions of interest in influenza and rotavirus indicates that our approach will be valuable in helping to identify regions in other viruses which warrant further investigation using structural approaches. Crucially this method removes a layer of subjectivity and also provides explicit ranges for the locations of these regions.

## Discussion

Here we have presented a new method for finding signals. Mathematically, the method finds regions within sequences of real numbers where the values are lower than expected. The benchmarking work above indicates that our method achieves the stated aims of (i) not requiring any input of order of length of the signal, (ii) outputs the location of the region (not just the indication of presence/absence of a region) and (iii) can find multiple separate regions in the same sequence. Our motivation is for application to genetic data, specifically seeking signals in virus sequences. However we envisage this method may be useful in many other contexts in bioinformatics and beyond.

For datasets that are relatively small, say hundreds or thousands in length such as with the viral sequences that motivated this work, our method is computationally inexpensive for single application, and we have presented approaches for speeding up the computation that will be of use when there are many or longer sequences to be analysed. However, benchmarking work to check the robustness of the method over many separate regimes is computationally demanding. The crux is the repeated reshuffles and recalculation needed in the bootstrap approach to the statistics. This could be obviated if there were some more analytic means of identifying when the largest value of *Z* is unusual or not. We have searched the statistical literature, but we cannot find exactly this problem being addressed, though the area of analysis of stochastic processes of ‘Brownian bridges’ has addressed some related problems [14]. The outline of the key remaining problem for this method is thus: suppose we have a random walk with steps drawn from some known distribution, then that random walk is linearly rescaled to make the start and end points match (and the step variance equal to one): what is the distribution of the largest *Z* in this case? Other statistical work relating to the properties of financial time series also seems relevant, as there may be some analogy in detecting ‘crashes’. However, we have not found anything that resolves our statistical questions: we are seeking the full extent of an unusual long downwards run, not just detecting a set drop over a short time or detecting the start of a run.

Considering previous approaches in signal processing, our proposed approach differs in that it is applied to the entire sequence of numbers rather than successively to small subsequences and that few assumptions are made about the characteristics of the signals. We have found no extant approach that has all the desired properties simultaneously. For example in image processing, region growing algorithms [15–20] do not assume that a given region has a particular size or that the magnitudes of the pixels it contains are predetermined, and allow a global comparison between regions. But in forming regions, these algorithms rely upon comparing pixels with other pixels defined by the algorithm to be ‘adjacent’. This definition introduces a length scale so that either similarity or graded variation at scales larger than the defined scale can be missed. Many region growing algorithms also are sensitive to the arbitrary initial choice of region from which to grow. Histogram segmentation techniques [21–23] use an entire dataset to produce a global histogram of values, from which criteria for classifying elements of the dataset are derived, but usually assume that the distribution will be bimodal and not multimodal (to avoid classifying values representing the edge between two non-adjacent modes into a third set), whereas our method can deal with signals of differing magnitudes. In addition, histogram techniques typically use information about nearby sequence elements on a fixed scale or do not make use of information about nearby sequence elements at all, whereas our method uses proximity but no pre-specified scale.

Sliding window, or moving average approaches, in which a point is replaced by the (possibly weighted) mean of neighbouring points, have been widely used in many fields with examples in geopositioning [24], finance [25, 26], clinical chemistry [27], epidemiology [28], physiology [29] and molecular biology [1, 30], amongst others, while in image processing a moving average approach using the median has been described [31]. These approaches allow comparisons to be made across the dataset, but the use of a window inherently imposes a length scale, maximising the signal-to-noise ratio of features whose size is similar to the window size. A similar issue limits the application of machine learning: the requirement for a training set needs *a priori* specification of the lengths and magnitudes of runs of low numbers either directly, or indirectly via already knowing characteristics of signals analogous to those being sought. For problems where the length of the features to be found is known, a moving average approach may be appropriate and indeed it may be possible to take advantage of multiple known length scales [32], and machine learning approaches may also be used, for example such as in transmembrane helix prediction [33]. However the danger is that smaller signals will be dismissed as noise and larger signals may also be missed, or their extent underestimated. For more general application where the size characteristics of the signals are unknown, our method removes the subjectivity of an *a priori* choice of length scale whilst allowing comparisons throughout a global dataset.

Our method finds signals (expressed as regions of low numbers in a sequence of real numbers) by making global comparisons across an entire dataset, in contrast with many signal processing methods that focus upon local comparisons. For the application that motivated development of our method (seeking signals in virus sequences), the size of the dataset is small enough to yield to current computing power, meaning a global comparison is both feasible and preferable. For datasets that are much larger than computationally tractable by our approach, some compromise will need to be made, for example to revert to moving averages with specified signal length scale.

Focussing on our specific application in viral genetics, the reader may ask why this naïve and almost model-free approach should be used over a more mechanistic phylogenetic model. With influenza packaging signals a tree-based approach was explored in some depth previously, using full genetic models for mutation at different sites, in an attempt to use the full information from inferred trees to detect packaging signals [34]. Owing to the extensive model development and computational needs this more advanced project took years longer to complete. The results were largely only marginally different from the original analysis using a naïve approach. Many established off-the-shelf evolutionary models output scores per site (which might be inferred substitution rates, say, using some codon evolution model), so a method for detecting regions is necessary in those cases. In addition, even if a complex population genetic model exists for the system under attention that builds the possibility of constrained regions into the evolutionary model, then we would still recommend also using the simple approach presented here: it is simple and fast. If it yields similar results to those using very detailed models, then it gives the reassurance that the results are likely to be robust to model assumptions. Our tree-independent approach may be particularly useful for viruses that have been relatively unstudied, where there is little historic sequence data, and also for viruses that recombine often, as well as other cases where phylogenetic models face particular difficulties.

The data explosion in biology and beyond has led to a plethora of new methods and software, but available computational tools are often very elaborate, tailored to purpose and often require large teams to use [35]. We have developed this method in response to our own research needs. We think this method is straightforward for a single researcher with basic programming skills to implement. The required computational power needed for viral bioinformatics with this method is likely within reach of all research groups. This method can be used *ad hoc*, or integrated into an analysis pipeline. We hope that this offering will enable new studies that were not previously feasible, and hence new discoveries to be made.

## Supporting information

S1 Appendix Detailed worked example. This gives a detailed worked toy example, where the dataset is small enough to give as explicit values. This example is also used to illustrate the approaches for computational speed improvements.
(PDF)

S1 Dataset The nMPD scores for 12 virus genes. These data are derived from previously published works, as described in the main text. They are in the correct form to apply the algorithm presented in this paper. The unzipped file name gives the virus and gene. The individual files then contain two columns: the first columns gives the value of the normalised mean pairwise distance (MPD) in codon order, and the second column indicates with 1 when that position should be excluded from analysis as it is uninformative (0 otherwise). The first columns are used as the values *x*_*i*_ in our algorithm, where *i* is the row number, excluding the uninformative positions.
(ZIP)

S1 File Implementation in Mathematica. This gives the code to run the algorithm in Mathematica (Wolfram Research, Inc). This was tested in Mathematica 11.0.1. The methods are given in both short form, and commented up, and two examples from data through to graphical output are given: for a simulated sequence and for a virus dataset. This latter example is set to import data in the form used in S1 Dataset.
(NB) Click here for additional data file.

S2 File Implementation in Python. This gives the code to run the algorithm in Python (Python Software Foundation).
(PY) Click here for additional data file.
