# Supplementary material for: A new method for detecting signal regions in ordered sequences of real numbers, and application to viral genomic data
Source: PLoS One. 2018 Apr 13;13(4):e0195763. doi: 10.1371/journal.pone.0195763 (PMC5898753; doi:10.1371/journal.pone.0195763)
Supplement: S1 Appendix — This gives a detailed worked toy example, where the dataset is small enough to give as explicit values. This example is also used to illustrate the approaches for computational speed improvements. (PDF) [file pone.0195763.s001.pdf]

A new method for detecting signal regions in ordered sequences, and application to viral genomic data

Julia R. Gog[1], Andrew M. L. Lever[2,3], and Jordan P. Skittrall[4]

[1]Department of Applied Mathematics and Theoretical Physics, University of Cambridge
[2]Department of Medicine, University of Cambridge
[3]Department of Medicine, National University of Singapore
[4]Cambridge University Hospitals NHS Foundation Trust, Cambridge

S1 Appendix: Detailed worked example

1 Introduction

This document gives a worked example of the application of the methods described in the main text. In the first section, we apply our method of finding maximum Z to a simple simulated dataset. In the second section we show how the statistical testing works for this example. In the final section, we use the same example to illustrate the methods discussed in the main text for improving the speed of computation.

2 Finding max Z

Our method is for finding low regions in an ordered sequence of numbers $x_1, \dots, x_n$. Here we illustrate this with a specific sequence of 24 numbers: $x_1, x_2 \dots, x_{24}$. These are first normalised by shifting by a constant to have mean zero and rescaling them by a (positive) constant to have variance one: $\hat{x}_1, \hat{x}_2 \dots \hat{x}_n$. Finally the partial sums of the $\hat{x}_i$ are formed to give the c_i. These are all given numerically in Table 1. These are also shown graphically in Figure 1. Note that plotting the c_i is often enough to reveal to the eye where low regions are likely to be, as it does here.

Next we find Z:

$$Z = \max_{i,j:i<j} Z_{ij}$$

where

$$Z_{ij} = \frac{c_i - c_j}{\sqrt{j-i}} \quad \text{for } 0 \leq i < j \leq n$$

As this toy example is small, it does not take long to search over all possible i and j, but computational speed improvements are discussed below. For this example, Z is maximised with $i = 8$ and $j = 16$. Note that this means that the positions of the original data included are 9 to 16 inclusive, discovered by checking carefully which x_i are the difference between c_8 and c_{16}. For this pair, $Z = 3.24$.

i	x_i	$\hat{x}_i$	c_i
0	-	-	0
1	5.627	0.509	0.509
2	5.173	0.233	0.742
3	5.945	0.702	1.443
4	6.208	0.862	2.305
5	6.342	0.943	3.248
6	4.624	-0.101	3.148
7	7.176	1.45	4.597
8	4.944	0.094	4.691
9	1.859	-1.781	2.911
10	4.061	-0.443	2.468
11	0.299	-2.728	-0.261
12	3.305	-0.902	-1.163
13	4.587	-0.123	-1.286
14	3.166	-0.987	-2.272
15	3.597	-0.725	-2.997
16	2.37	-1.47	-4.467
17	6.706	1.164	-3.303
18	6.752	1.192	-2.111
19	5.46	0.407	-1.703
20	5.47	0.413	-1.29
21	4.962	0.105	-1.185
22	4.449	-0.207	-1.392
23	6.023	0.749	-0.643
24	5.848	0.643	0.

Table 1: **Toy worked example values.** *The x_i are example data (given exactly), and the other columns follow our method as given in the main text (shown rounded to three decimal places).*

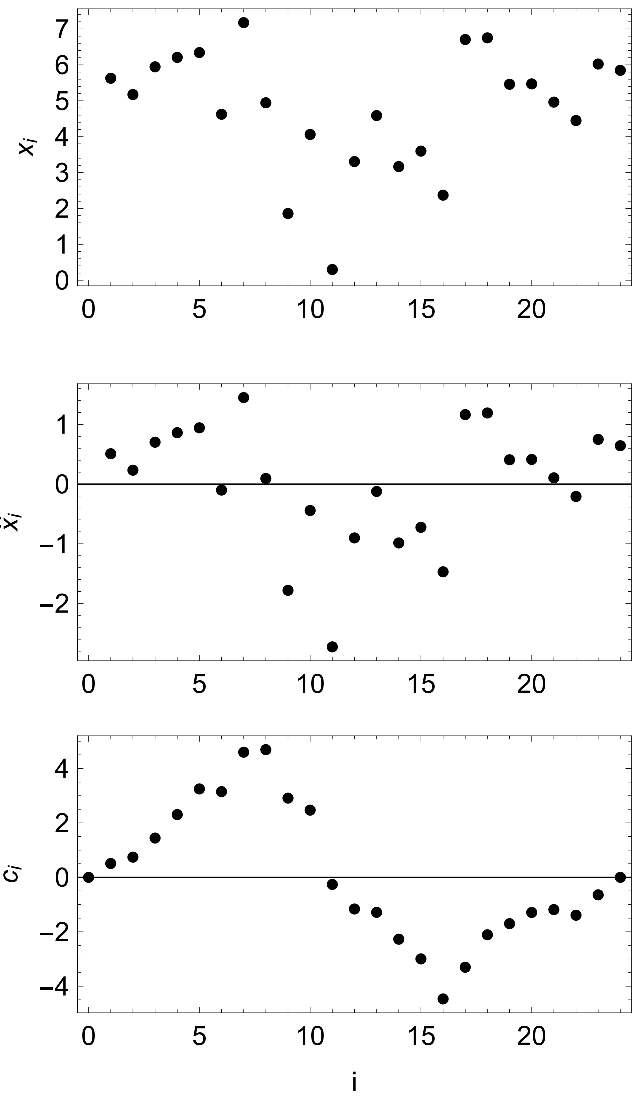

Figure 1: **Toy worked example plots.** *The upper panel shows the raw data* x_i*. The middle panel shows the normalised data* $\hat{x}_i$*: this is just a linear shift in the y-axis. The lower panel shows the accumulated normalised data* c_i*.*

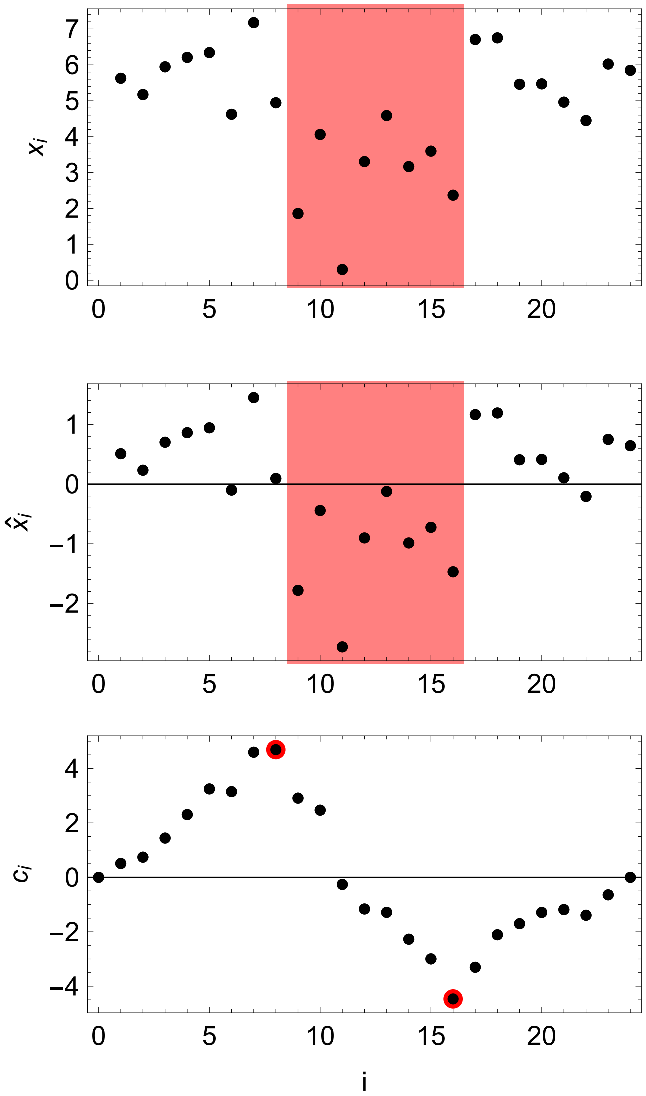

Figure 2: **With region marked.** *Similar to Figure 1, but with the region of interest marked in the top two plots, and the start and end points that give the largest* Z *in the bottom plot.*

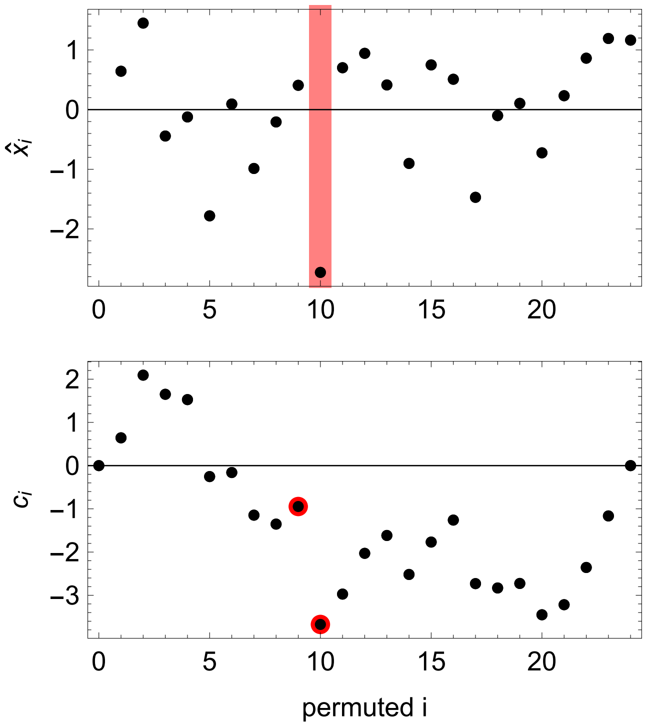

Figure 3: **Permuted data with region marked.** *Here the $\hat{x}_i$ are permuted and then the c_i recalculated.*

3 Statistical Testing

As described in main text, to determine if this 'real' Z is statistically significant, permute the original datapoints and find the new Z and do this repeatedly. As the normalisation step is the same no matter what order the x_i are in, it is slightly more efficient to start with the $\hat{x}_i$ and permute those with no further need to normalise. One example is given in Figure 3. In this case, the start and end point of the run giving maximal Z are consecutive, corresponding to a single point in the 'region' of interest. This example has $Z = 2.73$. In fact for *any* permutation, this will be a lower bound on the maximal Z as this same scoring possibility will be present (it is the largest single downwards step, and we we can read off Table 1 that this corresponds to $\hat{x}_{11}$). Indeed for this dataset, this often comes out as the maximal Z for a random permutation but not always (this is typical for a small dataset).

Computing the Z for 1000 random permutations of the data, we find $Z < 3.24$ in all but 17 runs. So we would say this is significant at 5% level (but not the 1% level). Hence our method would propose x_9 to x_{16} comprise an interesting region, unlikely to have occurred by chance. (Indeed, this happened to be exactly what was input in generating this example.)

To complete the method, remove x_9 to x_{16} and repeat the process for the remaining dataset. This is not shown here (as it runs through much the same) and in this the second proposed region is not statistically significant. So we end with the region 8–16 as the only significant one found.

4 Computational speed improvements

For finding the maximal Z_{ij}, one could naïvely compute Z_{ij} for all possible pairs of i, j with $i < j$. With n datapoints, this is $n(n-1)/2$ pairs for which to compute Z_{ij}. For the toy example with

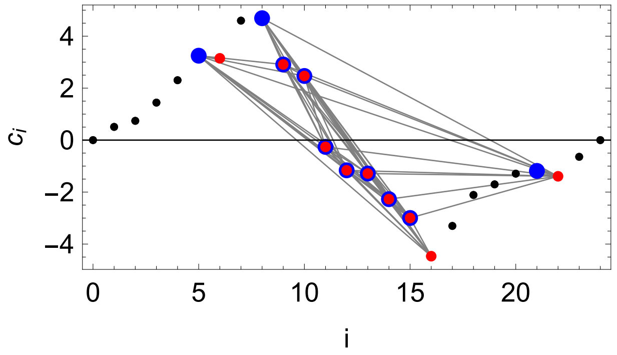

Figure 4: **Potential start and end locations.** *This is as Figure 1 lower panel, but with the potential starts marked in blue and potential ends marked in red (with some points marked both). The lines in grey connect each of the start-end pairs that occur in the right order (start first) - these are not all easy to see, but there are 55 of them.*

$n = 24$, this 276 pairs to check, which is not at all unreasonable, and indeed for n of hundreds or thousands, this does not take long to compute. The real computational cost comes in when bootstrapping using permuted datasets, as done above, where we need to repeat the whole process many times, say 1000 or 10,000 times. In the main text, some suggestions are given for approaches that will decrease the amount of computational work required by limiting the set of pairs i, j that need be checked: we illustrate them here with the example above.

The first relatively easy speed-up is to note that the best pair i, j must have the property that both the first step and the last step in the sequence $c_i, c_{i+1}, \ldots, c_j$ must be steps downwards. If not, if one of them is neutral or upwards, then trim it off: the resulting sequence will be shorter (the denominator $\sqrt{j-i}$ decreases), and have greater or equal height drop (the numerator $c_i - c_j$ does not decrease), hence the corresponding Z_{ij} will be larger, hence we did not have the maximal Z_{ij} originally.

The steps 'downwards' will correspond exactly to the values of i where $\hat{x}_i < 0$, so these i give the ends, and subtracting one from each gives the starts (given more formally in the main text). These are then a shortlist for the possible 'starts' for i and 'ends' for j: call these sets $s_1, s_2, \ldots, s_N$ and $e_1, e_2, \ldots, e_N$ respectively. Note that $s_u + 1 = e_u$. Pairing up each of the starts and ends that occur in the right order now, we have $(N+1)N/2$ pairs (as s_u can be paired with e_v for $u \leq v$).

Typically $N \approx n/2$ so the number of pairs needed to check will have reduced by a factor of four (from $n^2/2$ to $n^2/8$ to leading order). For the example here, actually only 10 of the 24 $\hat{x}_i$ are negative (skewed distribution, which can happen when there is a signal to be found) so we go from 276 pairs to only 55. The potential starts and ends for this example are shown in Figure 4. Note as the bootstrapped data are permutations of the $\hat{x}_i$, the number of pairs to compare will be the same for all of these (55 for this example).

It is possible to go further with this approach and limit in which combination these potential starts and ends may be paired up. This is described formally in the main text, but is slightly harder to visualise, so a specific example is picked out here. Focussing on the last potential end point (at $j = 22$), does it make sense to check the Z value for it paired with each of the start points? No: for all the start points apart from 21, they would achieve a greater Z with $j = 16$ as the end point. This is because $j = 16$ is *both* earlier and lower, giving a greater Z. This is illustrated in Figure 5. Indeed for each end point, we can identify whether there is another end point that 'beats' it in this way, and if more than one choose that which is closest, and then exclude all pairings between the original end point and any starts earlier than the other end point. The function f defined in the main text encapsulates this: $f(v)$ gives the index of the end

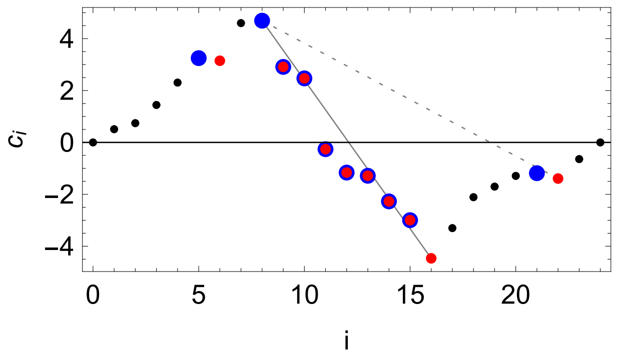

Figure 5: **Example of a pair that can be excluded** *For example, compare the pair marked by the dashed line (8 to 22) with the pair marked by the solid line (8 to 16): the solid line has smaller width $(j-i)$ and larger height $c_i - c_j$ so will certainly give a higher Z, so the dashed pair can safely be excluded. Indeed as the end point at 16 is lower than that at 22, we can exclude any pairings between 22 and starts which are before 16. (In this case, that leaves 21 to 22 as the only pair to check involving 22.)*

point that beats the vth endpoint (or zero if none). Thus we can exclude pairs $i = s_u$ and $j = e_v$ for $u \leq f(v)$, leaving just $f(v) < u \leq v$.

This extra method is actually not very helpful for our example here, as the endpoints are in almost decreasing order, and this will be at least partially the case if there is a real signal there to detect. In fact here, $f(v) = 0$ for all v except for the last, where $f(10) = 9$, which corresponds to the case illustrated in Figure 5. So indeed only these nine pairs are excluded and the number of pairs to check has merely gone down from 55 to 46.

But the real benefits from this method is not in calculating max Z for the real data, but for the bootstrapped permutations as the endpoint heights are not typically in such an order. Returning for example to the permutation in Figure 3, the same method here reduces the pairs from 55 to 31. Repeating over many permutations, the average number of pairs is around 28.

For this short example, the extra complication of computing f may not seem to be worth the relatively modest reduction in pairs that need their Z_{ij} computed. For clarity here then, two additional points are worth noting. Firstly, as we scale up n, the length of the input dataset, the benefit scales disproportionately: rather than the number of pairs to check growing as n^2, it appears to be growing rather more slowly. For example, for a sequence of independent identically distributed uniform random samples of length $n = 1000$, pairing all i and j gives 499,500 pairs to check. Limiting to potential starts and ends as described reduces this to around 125,000 pairs. The last step above of limiting which starts and ends can be paired together dramatically reduces this to about around 10,000 (based on numerical simulation). Secondly, the time taken to compute the f also scales less than quadratically with n using the iterative method given in the main text (and sample code), where later evaluations of f reuse previous values iteratively.

In summary, these additional computational speed-ups are not crucial for small datasets (say n in the order of tens), but these methods are offered and available in sample code, and are increasingly worth the complication with larger datasets.
